# Endocrine disrupting and carcinogenic effects of decabromodiphenyl ether

**DOI:** 10.3389/fendo.2023.1183815

**Published:** 2023-06-02

**Authors:** Yi Wang, Xinpei Wang, Shaofeng Sui, Zhiyan Liu

**Affiliations:** ^1^ Department of Pathology, Shanghai Sixth People’s Hospital Affiliated to Shanghai Jiao Tong University School of Medicine, Shanghai, China; ^2^ The Key Laboratory of Experimental Teratology, Ministry of Education and Department of Pathology, School of Basic Medical Sciences, Shandong University, Jinan, Shandong, China; ^3^ Department of Environmental Health, Division of Health Risk Factors Monitoring and Control, Shanghai Municipal Center for Disease Control and Prevention, State Environmental Protection Key Laboratory of Environmental Health Impact Assessment of Emerging Contaminants, Shanghai, China

**Keywords:** BDE209, endocrine disrupting, carcinogenesis, thyroid hormone, environmental exposure

## Abstract

**Background:**

Decabromodiphenyl ether (BDE209), an essential industrial flame retardant that is widely used, has recently been reported to be increasing in human serum. Due to the structural similarity between BDE209 and thyroid hormones, its toxic effects on the thyroid are of particular concern.

**Methods:**

Original articles in the PubMed database were collected using the terms “BDE209”, “decabromodiphenyl ether”, “endocrine disrupting”, “thyroid”, “carcinogenesis”, “polybrominated diphenyl ethers”, “PBDEs,” and their synonyms from inception up to October of 2022.

**Results:**

Of the 748 studies initially identified, 45 were selected, which emphasized the adverse effects of BDE209 on endocrine system. BDE209 may have a toxic effect not only on thyroid function but also on thyroid cancer tumorigenesis at multiple levels, such as by directly interfering with the TR, hypothalamic-pituitary-thyroid (HPT) axis, enzyme activity, and methylation. However, it is impossible to draw a definitive conclusion on the exact pathway of thyroid toxicity from BDE209.

**Conclusions:**

Although the toxic effects of BDE209 on the thyroid have been well investigated, its tumorigenic effects remain unclear and further research is necessary.

## 1 Introduction

Decabromodiphenyl ether (BDE209) is a member of the polybrominated diphenyl ether (PBDE) family. As the most common brominated flame retardant (BFR), it is widely used in industrial and daily essentials such as electronic equipment, building materials, and textiles. The demand and application of PBDEs in China are extensive, and the production hubs are concentrated in Shandong and Jiangsu. The consumption amount of BDE209 is more than 75% of the total BFRs (1, 2) because it is inexpensive and convenient. However, due to its lack of binding effects from chemical bonds, BDE209 easily escapes from the matrix material into the environment (3). It is fat-soluble and is widely detected in the atmosphere, water resources, soil, animals, and human bodies (4, 5). BDE209 was included as a persistent organic pollutant (POP) under the Stockholm Convention in 2009. The Chinese Ministry of Environmental Protection and the National Health Commission of China launched the List of Priority Control Chemicals (first batch) in 2017, which included BDE209 as a priority control chemical. Nevertheless, due to its stability and permanence in the environment, the risks to human health still remain. It is reported that residents of the producing area have serum concentrations of BDE209 as high as 3100 ng/g lipid weights (lw) (4), which is associated with diseases such as thyroid dysfunction. Therefore, it is urgent to investigate the toxic effects and potential mechanisms of BDE209 on the thyroid gland.

## 2 Materials and methods

### 2.1 Search strategy

The present systematic review was conducted according to the Preferred Reporting Items for Systematic Reviews and Meta-Analysis (PRISMA) guidelines for relevant studies published through October 2022 using the PubMed database (6). The search strategy was developed using the Medical Subject Headings (MeSH)-related terms/subheadings, using the terms “BDE209”, “decabromodiphenyl ether”, “endocrine disrupting”, “thyroid”, “carcinogenesis”, “polybrominated diphenyl ethers”, and “PBDEs”. The search strategy was modified for each database. Articles not written in English were excluded. Manual backward and forward citation searches were also conducted to ensure that no publications were missed.

### 2.2 Study selection

**Figure 1:**
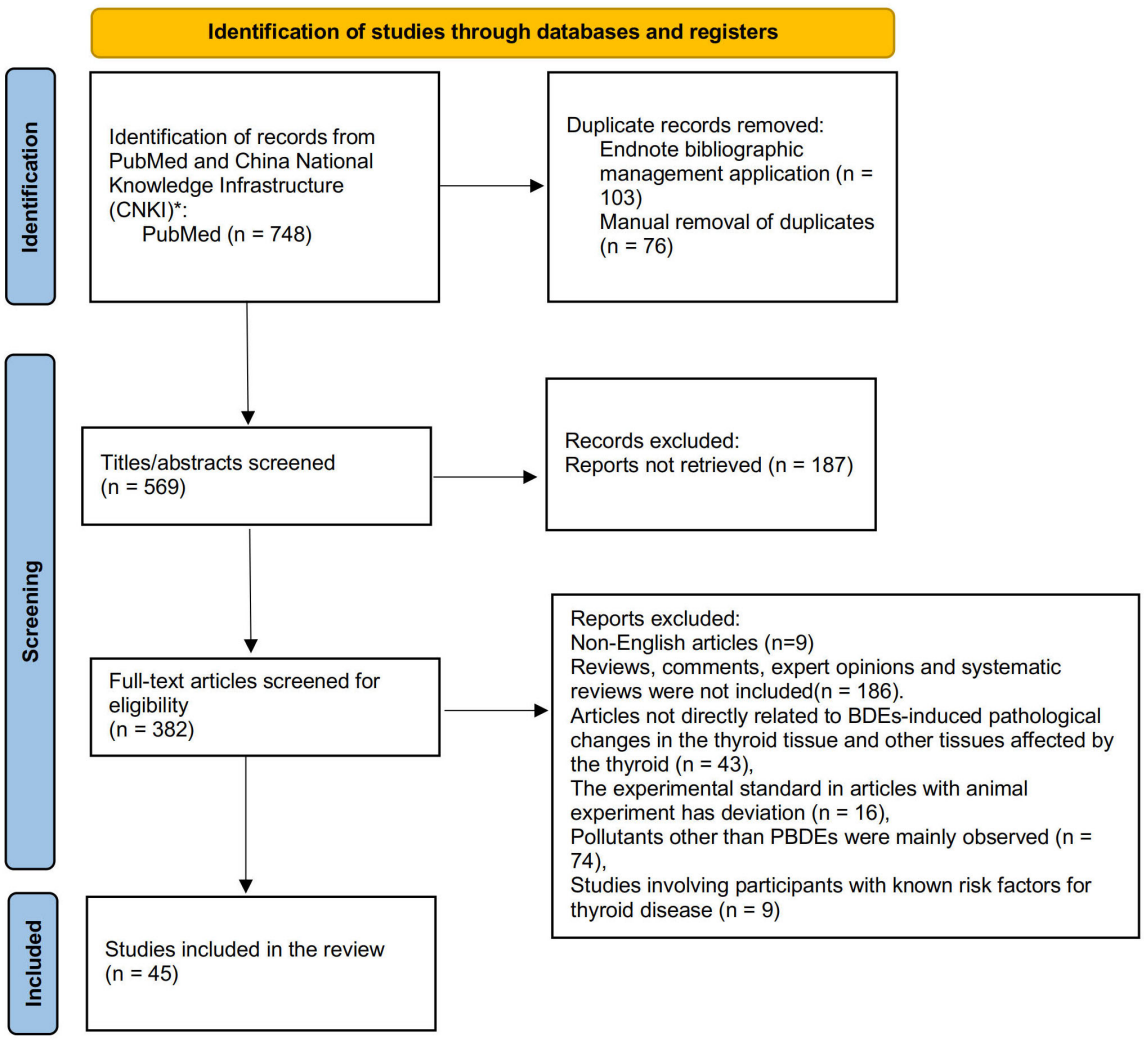
Article search and identification using PRISMA.

**Figure 1** depicts the results of the PRISMA search strategy. After duplicate reports were removed, 382 articles were identified. The inclusion criteria for this systematic review are as follows:

1. Original articles describing the effect of BDE209 exposure on thyroid tissue and thyroid-affected tissues (e.g., normal thyroid tissue, thyroid tumor tissue, or blood).

2. Thyroid function tests and/or at least one imaging technique (thyroid ultrasound or radionuclide study), cytological confirmation (fine needle aspiration), or medical certificate issued by a hospital were used to diagnose the impact on thyroid function.

3. Original articles describing the evidence and possible mechanism of the association between PBDEs (especially BDE209) and thyroid disease across studies and summarizing them at different levels (thyroid endocrine disturbance, potential carcinogenicity).

Reviews, comments, expert opinions, and systematic reviews were not included. The titles and abstracts of the retained publications were independently screened by two authors (YW and XW) to determine whether they met the criteria for inclusion. Following that, the authors (ZL and SS) screened the full texts of the retained publications to assess their eligibility for inclusion.

## 3 Results

Through a rigorous search of the PubMed database, a total of 45 studies emphasizing the adverse effects of BDE209 on thyroid function were identified. These studies were divided into two broad categories based on their methodology: epidemiologic studies and studies on hypothesized mechanisms. Epidemiologic studies were summarized in **Supplementary Table 1**. Studies on hypothesized mechanisms were summarized in **Supplementary Table 2**.

## 4 Exposure and bioconcentration of BDE209 in the environment

PBDEs have the general chemical formula C_12_H _(0–9)_Br_(10-1)_O and include 209 congeners. BDE209 is the most commonly used PBDE and has the highest bromine content.

BDE209 is more likely to be particle-bound in the atmosphere and has higher lipid solubility on the ground (7). This has drawn increasing attention to its toxicity to human health. In addition to air, BDE209 can be dispersed in the natural environment through the movement of water currents, sediments, and soil (8). Because of its lipid solubility, BDE209 accumulates easily in organisms and produces biomagnification along the food chain. In nature, zebrafish larvae exposed to BDE209 accumulated 10 times more BDE209 than the control group (9). Terrestrial biota have higher levels of BDE209 than aquatic biota.

There has been research on the correlation between BDE209 exposure and human serum levels. The closer to the electronic recycling and BDE209 manufacturing sites, the more severe the contamination is. Serum concentrations of BDE209 in occupational workers from a PBDE manufacturing plant ranged from 67.4 to 109,000 ng/g lw, with a median of 3420 ng/g lw, contributing to 93.1% of the total PBDEs (10). Hu et al. found that BDE209 was the highest measured amount (accounting for 73.6% of the total PBDEs) when they evaluated the levels of PBDEs in the human serum of adult residents along the Yangtze River (11). Sabrina et al. found that the geometric mean plasma concentration of BDE209 was the highest of the PBDE congeners (18 ng/g lw, geometric standard deviation [GSD]: 2.8) among workers exposed to electronic waste recycling in Canada – 10 times higher than the control group (12). Detection rates for BDE209 were slightly higher in women (13).

In the general population, ingestion is the main route of exposure to BDE209. For the population with occupational exposure, the main modes are inhalation and skin exposure. For infants, these pathways also include placental and breast milk exposure. The exposure level, body burden, and toxic effect were higher in infants than in adults (14).

It is noteworthy that many studies have shown that BDE209 can affect thyroid hormone homeostasis *in vivo* (15). Thyroid hormone (TH) is the essential hormone for growth, development, and reproduction. It is more susceptible to being influenced by exogenous interfering substances when the thyroid system is still developing in the early stages of life (16). Debarshi et al. indicated that BDE209 could affect the reproductive potential of the testis, a THs-responsive organ, by altering THs status (17).

## 5 Thyroid endocrine disruption by BDE209

As an endocrine-disrupting chemical (EDC), BDE209 may affect thyroid function in several ways (18). As a congener of THs, the connection between BDE209 and thyroid metabolism and diseases has become a hot topic. BDE209 was the only component that had a positive correlation to T3 (ß-coefficient 0.079) and was the relatively largest contributor (66% in ΣPBDE) (15, 19).

In the research on occupational workers, urine and serum concentrations of BDE209 were positively correlated with total thyroxine (TT4, r = 0.270, p = 0.029) and total triiodothyronine (TT3, r = 0.232, p = 0.061). A 10-fold increase in serum BDE209 was associated with a 7.8% increase in TT4 and a 5.4% increase in TT3 (10). However, there were no significant associations between BDE209 in human hair and nail samples and THs or thyroid antibodies, which suggests that sample type and sample size may lead to statistical differences.

In the resident study, no significant associations between PBDEs and thyroid function were observed in 36 anglers in the state of New York. However, Bloom et al. indicated that PBDEs may be positively associated with FT4 when the sample size is increased by approximately nine times (20). In a cross-sectional study of 85 Alaskan natives, there were no significant associations between serum levels of BDE209 and either free or total T4. However, they were significantly associated with THs when BDE47, BDE153, and BDE209 were covariates in the same model (13).

The meta-analysis shows that the relationship between PBDE exposure and thyroid function follows an approximately u-shaped curve. The low level was negatively correlated with THs, while the high level is positively associated with THs (21). The reason for this difference may be related to the sample type, sample size, and different exposure times and doses of BDE209. Future studies need to expand their scope and study population for prospective longitudinal studies and in-depth analysis.

## 6 Endocrine disruption mechanisms of BDE209

### 6.1 Effects of BDE209 on the HPT Axis

BDE209 and its metabolites have chemical structures that are similar to those of T3 and T4, so they can bind with thyroid hormone receptors (TRs), thyroid binding globulin (TBG), transthyretin (TTR), and related enzymes. These interactions can have different effects on the synthesis and transport of THs, the accurate regulation of the hypothalamic-pituitary-thyroid (HPT) axis, and thyroid function. The HPT axis is a complicated negative feedback regulation system. Thyrotropin Releasing Hormone (TRH), which is produced by neuroendocrine cells in the hypothalamus, promotes the synthesis and release of thyroid-stimulating hormone (TSH) and THs (T4 and T3). Chevrier et al. found that there was a negative correlation between serum PBDEs and TSH during pregnancy, and the incidence of subclinical hyperthyroidism increased significantly, which may indirectly influence fetal growth (22).

### 6.2 Effects of BDE209 on thyroid function

Based on animal model studies, BDE209 produces direct and indirect toxic effects on the thyroid gland, and its endocrine-disrupting mechanism may be related to oxidative stress and enzyme activity (23). Higher doses of BDE209 may cause the reduction of thyroglobulin (TG) and thyroid peroxidase (TPO) by the atrophy of the rough endoplasmic reticulum or down-regulation of related genetic alterations (24). Further research showed that PBDEs increased thyroglobulin antibodies and TPO-Ab in the female population (r = 0.453, p = 0.045) (10, 25).

### 6.3 Effects of BDE209 on thyroid hormone conjugated enzyme

Although PBDEs can conjugate with enzymes involved in the synthesis and metabolism of THs, different congeners may have different substrate metabolic activities. The higher the brominated PBDEs, the stronger the binding affinities with TR and TTR (26). Therefore, BDE209 and its metabolites may disrupt TH homeostasis through strong competitive binding to thyroid hormone transporters (TBG and TTR) to increase fT4 and further reduce T4 half-life *in vivo* (27, 28). PBDEs can induce UDP-glucuronosyltransferases (UGTs) in the liver and eliminate thyroxine through the bile to cause THs dysfunction. Chen et al. showed that the decreased TT4 level and the increased TT3 level are accompanied by decreased UGT gene transcription after BDE209 exposure (29). Moreover, BDE209 can also conjugate with TR and competitively inhibit THs (30).

### 6.4 Effects of BDE209 on TH activation

Several animal studies show that BDE209 exposure can regulate the expression or activity of thyroid hormone deiodinases. BDE209 induced upregulation of deiodinase type I (Dio1) activity in human hepatocytes (31) and deiodinase type II (Dio2) activity in larvae (32). However, PBDEs down-regulated Dio2 activity in human glial cells (33). Long-term dietary exposure to BDE209 could cause significant reductions in T4 outer ring deiodination (T4-ORD) and inner ring deiodination (T4-IRD) (34). Qin et al. suggested that BDE209 reduces the expression of the deiodinase type III (Dio3) gene through induction of IG-DMR hypermethylation of the parent substance and inhibition of miR409-3p and miR668-3p expression. Reduced expression and insufficient activity of placental Dio3 lead to the direct exposure of fetal tissues to high levels of maternal active thyroid hormone, which endangers fetal brain development. This toxicity may persist into late fetal growth and early childhood.

BDE209 may enhance the absorption of heavy metals such as lead in earthworms and zebrafish, which would have a synergistic effect on the homeostasis of thyroid hormones. As a result, the comprehensive effect of BDE209 and heavy metals on the thyroid is more complicated and harmful (35–37).

### 6.5 Effects of BDE209 on thyroid inflammation

The research found that 31% of subjects who had been exposed to PBDEs had higher levels of thyroglobulin antibodies. Abnormal values in thyroglobulin antibodies could be seen in 80-90% of patients with chronic autoimmune thyroiditis and 50-60% of patients with Graves’ disease (25). Therefore, PBDE exposure may be a potential cause of susceptibility to autoimmune thyroid disease.

Different immunologic toxic effects of BDE209 were identified in 2021 (1): atrophy of immune organs (thymus and spleen); hepatomegaly accompanied by increased aspartate aminotransferase and oxidative stress; (2) changes in humoral (immunoglobulins) and cellular (lymphocyte proliferation and cytokine secretion) indices of immunity; (3) dose-dependent alterations in gene expression, leading to Th1/Th2 imbalance (38).

## 7 Potential carcinogenicity of BDE209

Many case-control studies have recently shown that BDE209 has potential carcinogenicity in biological matrices (39).

The National Toxicology Program (NTP) established an animal model to examine the toxicity of BDE209 in 1986. B6C3F1 mice (50 males and 50 females) were fed diets supplemented with different doses of BDE209 (25 and 50 g/kg) for 103 weeks. Compared to the control group, abnormal proliferation of thyroid follicular cells was found in the female and male groups, and the ratio of follicular thyroid adenomas increased (40). In 2011, Noyes et al. found that long-term dietary exposure to BDE209 caused cellular and structural atypia in thyroid follicles (16). Therefore, BDE209 may cause abnormal thyroid proliferation and thyroid follicular adenoma.

In 2017, Hoffman et al. found that the concentration of BDE209 in indoor dust was significantly associated with the prevalence of papillary thyroid carcinoma (PTC). Participants with above-median BDE209 exposure had a 2.29-fold increased risk compared to those with low BDE209 exposure. High concentrations of BDE209 were significantly associated with PTC with BRAF wild-type (14.2 times, p<0.05) compared to the control group (41). Deziel et al. showed a negative correlation between BDE209 and the pathogenesis of thyroid cancer in 2018 (42). However, several recent studies offer a different opinion. In 2021, Zhang et al. found that BDE209 was positively correlated with the risk of thyroid cancer, and its co-exposure with heavy metals aggravated the risk of developing it (43).

The above studies suggested the potential toxicity and tumorigenic effects of BDE209 on the thyroid gland. The tumorigenic effects may be related to exposure levels, time, methods, and the types of experimental animals used.

## 8 Study of the carcinogenic mechanisms of BDE209

**Figure 2:**
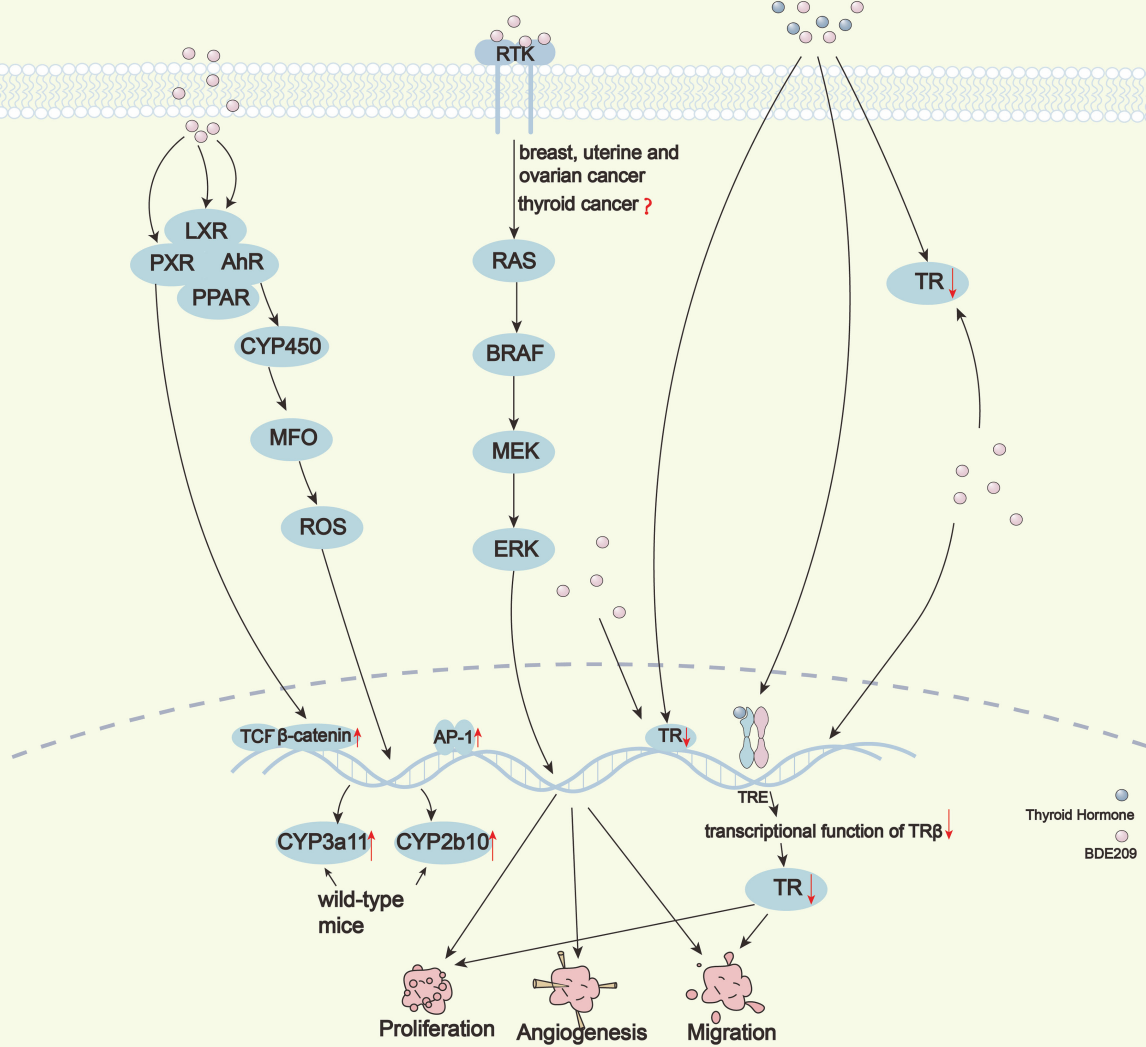
Hypothesis of the tumorigenic effect of BDE209 on thyroid cancer.

Although it has been widely reported that BDE209 induces thyroid cancer, studies on its potential carcinogenic mechanisms are relatively scarce. In a study using RNA-seq of thyroid tissue samples exposed to BDE209, 501 differentially expressed genes (DEGs) were screened out. After enrichment analysis on DEGs, it was found that BDE209 response-related genes can be significantly enriched in multiple KEGG and GO databases (44). The carcinogenic mechanism of BDE209 is multifaceted and relatively complex (**Figure 2**).

### 8.1 Interaction between BDE209 and transcription factors

Natàlia et al. predicted the proteins with which BDE209 may interact by computer target analysis. BDE209 could activate five types of key human transcription factors, such as the aryl hydrocarbon receptor (AhR), peroxisome proliferator response element (PPRE), TCF/ß-catenin, AP-1, and Oct-MLP (9). The low concentration of BDE209 can significantly activate AhR (45); however, different results have also been reported (46). BDE209 can activate the peroxisome proliferator-activated receptor (PPAR) pathway through indirect binding to lipid peroxidation-related receptors (47). High levels of BDE209 can activate TCF/ß-catenin. and AP-1 (46). BDE209 can affect the POU domain family of Oct-MLP, and these proteins are developmental regulatory factors of conservative evolution. These results can explain the carcinogenicity of BDE209 to a certain extent.

### 8.2 Inhibitory effect of BDE209 on thyroid hormone receptor

Thyroid hormone receptors (TRs) are ligand-dependent transcription factors that mediate the transcription of human growth- and development-related genes. PBDEs can bind to TRs due to the similarity of their chemical structures to THs, and the affinity increases significantly with increasing bromination. BDE209 has the highest degree of bromination and the strongest affinity with TRs among PBDEs (26, 48, 49). BDE209 may dissociate the DNA binding domain of TRs from thyroid hormone response elements (TRE) by competitively combining TRß with TH and then destroying TRß-mediated transcription (50). Recently, our research group demonstrated that BDE209 not only inhibits the transcriptional function of TRß but also directly blocks its gene expression, which then significantly promotes the proliferation of PTC cells both *in vivo* and *in vitro* (30). BDE209 may promote malignant transformation of thyroid follicular cells by inhibiting TRß at multiple levels, as supported by analysis of multiple public datasets (51).

### 8.3 BDE209-mediated thyroid injury through induction of oxidative stress

Reactive oxygen species (ROS) play an important role in cell damage and other aspects. When ROS in cells exceeds the capacity of antioxidant mechanisms, it is called oxidative stress. High levels of ROS may also activate signaling molecules as second messengers in intracellular signaling pathways that regulate cell proliferation and apoptosis (52). BDE47 has been shown to induce DNA damage *via* regulation of the ROS-induced oxidative stress response to mediate hepatocyte toxicity (53). Therefore, PBDEs could induce tissue damage and carcinogenicity through oxidative stress.

### 8.4 Upregulation of cytochrome genes induced by BDE209

Cytochrome is one of the important enzymes in mixed functional oxidase (MFO). It could form ROS complexes with molecular oxygen and oxidize into exogenous chemicals. In the cytochrome family, CYP2 and CYP3 are the main isoenzymes responsible for metabolizing drugs. Pacyniak et al. proved for the first time that BDE209 was an activator for exogenous nuclear receptors and that BDE209 could induce up-regulation of CYP3a11 and CYP2b10 mRNA levels by activating PXR in wild-type mice (54). Also, BDE209 can bind to AhR to regulate the function of downstream CYP450 (9). The abnormal expression of the CYP450 gene is associated with many types of tumors, including thyroid cancer. Furthermore, CYP450 can activate the oxidative metabolism of MFO to produce more ROS and oxidative stress, thereby increasing toxicity and becoming mutagenic or carcinogenic.

### 8.5 MAPK/PI3K pathways activated by BDE209

Thyroid cancer is usually driven by a few mutually exclusive somatic gene mutations or fusions involving the MAPK/PI3K pathways (55–58). Li et al. showed that BDE209 increases the incidence of breast, uterine, and ovarian cancers through the activation of the MAPK signaling pathway (59). Wang et al. found that BDE47 participated in regulating cell proliferation and was related to carcinogenesis in HepG2 cells through the DNA-PKcs/Akt pathway (60). However, it remains unknown whether BDE209 is involved in the pathogenesis of thyroid neoplasms through the MAPK/PI3K pathways.

## 9 Discussion

Because of its widespread use, common exposure, persistence, and bioaccumulation in the environment, the burden level of BDE209 in organisms is continuously increasing. Low-dose, long-term exposure to environmental BDE209 may pose an enormous threat to health. Moreover, the chronic toxic effect of BDE209 may be more severe in those subjects who are more sensitive to TH disruption, such as pregnant women and people with hyperthyroidism. Therefore, additional research is necessary to reveal its toxic dose and mechanisms of action.

Furthermore, as an endocrine pollutant, the relationship of BDE209 with thyroid diseases and thyroid cancer has been reported many times, but research is scarce on its carcinogenicity and its potential mechanisms in humans. Limited exposure studies of human environmental pollutants usually evaluate the effects of high-dose and short-term exposure, and it is difficult to obtain long-term, low-dose experimental exposure data.

BDE209 is easily metabolized into low-brominated diphenyl ethers and OH-PBDE, which could be affected by measurement error sources, including sample environmental contamination risk and analytical degradation risk. The features of environmental exposure in real life are the coexistence of multiple pollutants to produce synergistic or antagonistic toxicity, which causes much more obvious toxicological effects and more complicated mechanisms. THs are inversely associated with low- and high-brominated congeners, which may cause result errors. To provide a sufficient basis for the revision of the use standard for BDE209 products and the formulation of environmental pollution policy, further research is needed to standardize multiple risk factors, expand study sites, and study populations for in-depth analysis.

## Author contributions

Conceptualization, ZL, and SS; methodology, ZL; software, YW and XW; validation, ZL, and SS; formal analysis, ZL; investigation, SS; resources, XW; data curation, YW and XW; writing—original draft preparation, YW and XW; writing—review and editing, YW, XW, and ZL; visualization, ZL, and SS; supervision, ZL; project administration, ZL; funding acquisition, ZL. All authors read and approved the published version of the manuscript. All authors contributed to the article and approved the submitted version.
