# Supplementary material for: Endocrine disrupting and carcinogenic effects of decabromodiphenyl ether
Source: Front Endocrinol (Lausanne). 2023 Jun 2;14:1183815. doi: 10.3389/fendo.2023.1183815 (PMC10272517; doi:10.3389/fendo.2023.1183815)
Supplement: Supplementary file 1 [file Table_1.docx]

Supplementary Material

Endocrine Disrupting and Carcinogenic Effects of Decabromodiphenyl Ether

Yi Wang ^1†^, Xinpei Wang ^1,2†^, Shaofeng Sui ^3^, Zhiyan Liu ^1*^

*** Correspondence:** Zhiyan Liu: zhiyanliu@shsmu.edu.cn

# Supplementary Tables

**Table 1.** Human studies on the toxicity of PBDEs to thyroid

| **Authors/**  **Year** | **Age/Gender** | **Exposure** | **Study Aim** | **Main Findings** |
| --- | --- | --- | --- | --- |
| Xinhu Bi(5)  2007 | 18-81 | PBDEs, PCBs, OCPs | To determine the human body burden of PBDEs, PCBs and OCPs. | PBDEs accounted for 46% in samples from Guiyu, which was higher than previously reported in occupationally exposed populations. Serum PBDE concentrations did not correlate with PCBs or OCPs. The levels of PBDEs in individuals from Haojiang are possibly related to the recycling activity at Guiyu, through atmospheric transport. |
| Michael Bloom(20)  2008 | 29-45  Median age 39.5  M 30  F 6 | PBDEs | To generate hypotheses regarding associations between body burdens of PBDEs, received from environmental sources, and biomarkers of thyroid function. | No significant associations were observed between congeners, or their sum (PBDEs), and thyroid function. But, additional studies increasing sample size are needed. |
| Mary E. Turyk(25)  2008 | 30-82  Mean age 59  M 405 | PBDEs, PCBs, DDE | To determine whether PBDE body burdens are related to thyroid and steroid hormone levels, thyroid antibodies, and thyroid disease in a cohort of frequent and infrequent adult male sport fish consumers. | PBDE exposure, at levels comparable with those of the general U.S. population, was associated with increased thyroglobulin antibodies and increased T4 in adult males. |
| Jonathan Chevrier(22)  2010 | 18-45  M 270 | PBDEs | To determine whether PBDE serum concentrations are associated with  TH levels in pregnant women. | Exposure to PBDEs is associated with lower TSH during pregnancy. Findings may have implications for maternal health and fetal development. |
| Songie He(4)  2013 | 20-84  M164  F141 | PBDEs, DP, TBPH | To assess the PBDE concentration trend, and determine the concentrations of the emerging flame retardants DP and TBPH. | There was no change in the ∑_8_PBDE concentration order of magnitude between 2007 and 2011 in the serum of residents of the south coast of Laizhou Bay, China, but the relative contribution of BDE209 to ∑_8_PBDE was higher. |

**Table 1.** *Cont*.

| **Authors/**  **Year** | **Age/Gender** | **Exposure** | **Study Aim** | **Main Findings** |
| --- | --- | --- | --- | --- |
| Feifei Huang(19)  2014 | 19-55  Mean age 33.4  M60  F64 | PBDEs | To determine the human body burden of PBDEs and clarify the relationships between PBDEs and TH in the general population in Northern China. | BDE209 was the most abundant congener. The total PBDE concentrations in men were significantly higher than women. There were apparent correlations between concentrations of several PBDE congeners and TH. T3 was correlated with BDE99 and BDE209. No correlation between FT4 and PBDEs was observed. |
| Briseis Aschebrook-Kilfoy(39)  2015 | Mean age 61.8  M 35%  F 65% | PBDEs | To investigate whether serum concentrations of PBDE were associated with thyroid cancer. | The study does not support an association between exposure to PBDEs and thyroid cancer. |
| Kate Hoffman(41)  2017 | 26-80  Mean age 48  M 30  F 110 | PBDEs, FRs | To test the hypothesis that higher exposure to FRs is associated with increased odds of PTC. | Exposure to FRs in the home, particularly BDE209 and TCEP, may be associated with PTC occurrence and severity. |
| Lingchuan Guo(15)  2018 | 9-12  Mean age 10  M 93  F 81 | PBDEs, 8 species of NFRs | To examine the associations between PBDEs, NFRs and TH. | In contrast to ΣPBDE that was positively associated with T3 level, ΣNFR was not statistically associated with TH. ΣPBDE+NFR was significantly associated with T3 level. |
| Samuel C. Byrne(13)  2018 | 18-45  Median age 29  M 38  F 47 | PBDEs | To assess the relationship between serum PBDEs and thyroid function in a remote population of St. Lawrence Island Yupik. | Serum concentrations of BDE28/33, 47, and 100 were positively associated with TSH and FT3. Both BDE47 and BDE153 remained significantly associated with THs when BDE47, BDE153, and BDE209 were covariates in the same model. There were no significant relationships between serum PBDEs and either FT4 or TT4. |

**Table 1.** *Cont*.

| **Authors/**  **Year** | **Age/Gender** | **Exposure** | **Study Aim** | **Main Findings** |
| --- | --- | --- | --- | --- |
| Tian Chen(10)  2018 | 25-55  Mean age 34.5  M 43  F 29 | PBDEs | To investigate the relationship between BDE209 exposure and TH in occupational workers from a deca-BDE manufacturing plant. | The concentration of BDE209 in urine was highly correlated with that in the serum, indicating that urine may be a good non-invasive biomonitoring medium of BDE209 body burden in occupational workers. BDE209 in the serum was significantly and positively correlated with TT4 and marginally, positively correlated with TT3 in all occupational workers. |
| Nicole C. Deziel(42)  2019 | 21-84  M 500 | PBDEs, PBB | To investigate the relationship between PBDE/PBB exposure and PTC. | The results using single and multi-pollutant modeling do not support a positive association with PBDE/PBB and PTC risk. |
| Sabrina Gravel(12)  2020 | ≥18  M 77  F 23 | PBDEs, OPE, heavy metals | To measure biological concentrations of OPE metabolites, PBDEs, mercury, lead and cadmium in e-recycling workers, and to explore associations with thyroid and sexual hormones. | E-recycling workers had higher concentrations of BDE209, all OPE metabolites, and lead than commercial recycling workers. In men, a two-fold increase in BDE209 was associated with 3.1% higher levels of total T4. |
| Qian Zhang(43)  2021 | 32-60  M 84  F 532 | PBDEs, heavy metals | To examine the associations of TC risk with exposure to PBDEs and four heavy metals. | PBDE congeners (BDE28, BDE47, BDE99, BDE183, BDE209) were positively correlated with TC risk. Co-exposure to BDE209 and Pb had a negative interaction effect on TC risk. |
| Mingjun Hu(11)  2021 | 18-83  Mean age 52.5  M 70  F 259 | PBDEs, 14 urinary metals | To determine the levels of plasma PBDEs and urinary metals and evaluate the associations of co-exposure to both with THs among adult residents along the Yangtze River, China. | The burden of PBDEs and some heavy metals levels seems to be gradually accumulating among the rural residents along the Yangtze River. There was no statistically significant association of TSH with plasma PBDEs and urinary metals in current study. |

**Table 2.** Studies on hypothesized mechanisms of the toxicity of PBDEs

| **Authors/**  **Year** | **Animal** | **Exposure** | **Exposure Concentration** | **Main Findings** |
| --- | --- | --- | --- | --- |
| NTP(40)  1986 | F344/N rats and B6C3Fl mice | BDE209 | 0, 25,000, and 50,000 ppm (103 weeks) | By 2-year feed of BDE209, there was some evidence of carcinogenicity. Several non­-neoplastic lesions were observed at increased incidences, the most notable being thyroid gland follicular cell hyperplasia. |
| Ilonka A. T. M. Meerts(48)  2000 | / | PBDEs, PBP, TBBPA | 1.95 to 500 nM | Brominated flame retardants are very potent competitors for T4 binding to human TTR in vitro and may have effects on thyroid hormone homeostasis in vivo comparable to the thyroid-disrupting effects of PCBs. |
| David J. Brown(45)  2004 | mouse hepatoma cells | BP4A, PHT4, BP6, BDE209 and possible contaminants of BFR mixtures | 0.001nM-0.1mM | The results presented here demonstrate the ability of brominated flame retardants to activate the AhR signal transduction pathway at moderate to high concentrations as assessed. |
| Timo Hamers(27)  2006 | / | PBDEs | / | Antiandrogenic, antiprogestagenic, (anti-)estrogenic, (anti–)dioxin-like, and T3-antagonistic potencies of BFRs were demonstrated at the receptor level. |
| Krishna Das(61)  2006 | porpoises | PCBs, PBDEs, DDT, and DDE | / | The hypothesis that PCBs, PBDEs, DDT, and DDE may interfere with the harbor porpoise thyroid functions leading to severe interfollicular fibrosis still cannot be rejected. |

**Table 2.** *Cont*.

| **Authors/**  **Year** | **Animal** | **Exposure** | **Exposure Concentration** | **Main Findings** |
| --- | --- | --- | --- | --- |
| Erik K. Pacyniak(54)  2007 | C57BL/6 mice | BDE47, BDE99 and BDE209 | 100μmol/kg/day(4 days) | PBDEs induce cyp3a11 and 2b10, but not cyp1a1/2 in mouse livers. PBDEs activate both PXR and SXR in vitro. |
| Li-Ho Tseng(35)  2008 | CD-1 mouse | BDE209 | 10, 500, and 1500 mg/kg/day (17 days) | T3 was found to have decreased significantly in offspring. Histopathological examination revealed that prenatal exposure of PBDE 209 might be related with cell swelling of hepatocytes in male offspring. |
| Chris E. Talsness(62)  2008 | pregnant Wistar rats | BDE47 | 140, 700 μg/kg bw(until 27 days postpartum) | Administration of BDE47 at doses relevant to human exposure led to changes in the rat female reproductive system and thyroid gland. |
| Leo T.M. Van der Ven(8)  2008 | Rats | DecaBDEE | 0–0/0–1.87–3.75–7.5–15–30–30/30 mg/kg bw/day (28 days) | Most sensitive effect in males were increased weight of seminal vesicle/coagulation gland and increased expression of hepatic CYP1A and CYP2B.In females the most sensitive effect was decreased activity of CYP17 in adrenals. DecaBDE may represent an as yet unreported hazard for reproductive health. |
| Heather M. Stapleton(31)  2009 | human liver cells | BDE99, BDE209 | 10μM (24-72 hours) | Up-regulation of genes encoding for CYP1A2, CYP3A4, deiodinase type 1, and glutathione S-transferase M1 in hepatocyes exposed to both BDE99 and BDE209. |
| Suping C. Huang(47)  2010 | mouse cerebellar granule neurons | BDE47, BDE99, BDE100, BDE153 and BDE209 | 0-50nM | All BDE congener decreased cell viability and induced apoptotic cell death and also exhibit the same general mode of action and that the ability of each isomer to elicit such effects is dependent upon accumulation in neurons. |

**Table 2.** *Cont*.

| **Authors/**  **Year** | **Animal** | **Exposure** | **Exposure Concentration** | **Main Findings** |
| --- | --- | --- | --- | --- |
| Pamela D. Noyes(16)  2011 | fathead minnows | BDE209 | 9.8±0.16mg/g of food at 5% of their body weight per day (28 days) and depuration period (14 days) | Rates of T4-ORD and T4-IRD were reduced, which were remained after depuration period. Histological examination showed significantly increased thyroid follicular epithelial cell heights and vacuolated hepatocyte nuclei. These results suggest that juvenile fish may be susceptible to thyroid disruptors like PBDEs. |
| Jing An(53)  2011 | L02 cells | BDE47, BaP | BDE47(5, 10mol/L), BaP(50 mol/L) | BDE47 and BaP had a synergistic effect on oxidatively generated DNA damage via regulation on the oxidative stress response and the expression of CYP1 metabolism enzymes. |
| Kingsley Ibhazehiebo(50)  2011 | CV-1 monkey fibroblast-derived cells | BDE209, BDE47, DE71, 4’-OH-BDE49 | To examine the associations of TC risk with exposure to PBDEs and four heavy metals. | Several PBDE congeners may disrupt the TH system by partial dissociation of TR from TRE acting through TR-DBD and may disrupt normal brain development. |
| Wei Li(32)  2011 | larvae and adult rare minnow (Gobiocypris rarus) | BDE209 | 0.01, 0.1, 1, and 10 lg/L (21 days) | BDE209 may pose threat to normal thyroid and reproductive function in fish. |
| Marijana ĆURČIĆ (36)  2012 | Wistar rats | Cd, BDE209 | Cd(2.5, 7.5 and 15 mg/kg) and BDE209(1000, 2000 and 4000mg/kg)(28 days) | The Cd+BDE209 mixtures more potently disrupt thyroid hormone homeostasis than would be expected from these chemicals alone. |

**Table 2.** *Cont.*

| **Authors/**  **Year** | **Animal** | **Exposure** | **Exposure Concentration** | **Main Findings** |
| --- | --- | --- | --- | --- |
| Qi Chen(29)  2012 | zebrafish | BDE209 | 0, 0.08, 0.38, 1.92mg/L (until 14 days post-fertilization) | The exposure resulted in alterations of T3 and T4 levels, indicating thyroid endocrine disruption. The genes related to thyroid function were transcriptionally significantly changed. |
| Liulin Wang(60)  2012 | human hepatoma HepG2 cells | BDE47 | 10^−10^, 10^−9^ and 10^−8^ M | BDE47 has a hormesis effect in HepG2 cells and DNA-PKcs/Akt pathway may be involved in regulation of cell proliferation and apoptosis. |
| Zhi‑Hua Li(59)  2012 | multidrug‑resistant breast cancer cells, cervical cancer cells, ovarian cancer cells, and normal CHO cells | BDE209 | 5, 15, 25, 50, or 100nM | BDE209 can promote proliferation of various cancer cells from the female reproductive system and normal ovarian CHO cells. It also reduced tamoxifen, PKCα, and ERK inhibition‑induced apoptosis, and up‑regulated phosphorylation of PKCα and ERK1/2 proteins. |
| Xiao-Min Ren(26)  2013 | / | PBDEs | / | OH-PBDEs bound to TR with potency that correlated to their bromination level. OH-PBDEs have different activities on TR (agonistic or antagonistic), possibly due to their different binding geometries with the receptor. |
| Natàlia Garcia-Reyero(9)  2014 | zebrafish | BDE209 | 12.5mg/kg (8 days) | BDE-209 impacted expression of neurological pathways and altered behaviorof larvae. BDE-209 activated human aryl hydrocarbon receptor, peroxisome proliferator activating receptors, CF/b-cat, activator protein 1, Oct-MLP, and ERRα receptor in cell-based assays. BDE209 also inhibited human acetylcholinesterase activity. |

**Table 2.** *Cont*.

| **Authors/**  **Year** | **Animal** | **Exposure** | **Exposure Concentration** | **Main Findings** |
| --- | --- | --- | --- | --- |
| Wei Zhang(37)  2015 | earthworm (Eisenia fetida) | Pb, BDE209 | Pb(250μg/g) and BDE209(1, 10 and 100μg/g)(48 hours) | The toxic effects of Pb and BDE209 were synergistic |
| Simon Roberts(33)  2015 | human astrocytes | PBDEs, OH-BDEs | up to 5μM | Reductions in DIO2 activity caused by exposure to PBDEs may play a role in the neurodevelopmental deficits caused by these toxicants. |
| Min-Chul Lee(63)  2016 | marine copepod Paracyclopina nana | BDE47 | 0.1, 1, and 10 μg/L | BDE47 induced oxidative stress mediated activation of ERK and JNK signaling cascades in MAPK pathways. Activated MAPK pathways induced signal molecules that bind to TFs responsible for lipogenesis to EcR, SREBP, ChREBP promoters. |
| Yuwei Wang(24)  2019 | Male rats | DBDPE, BDE209 | 5, 50, 500 mg/kg bw/day (28 days) | BDE209 and DBDPE affected the expression of HPT axis related genes, which suggested that both their exposure could disrupt thyroid function in the direction of hypothyroidism and the underlying mechanism was likely to be oxidative stress and perturbations of HPT axis. |
| Debarshi Sarka(23)  2019 | mice | BDE209 | 500 or 700 mg/kg (28 days) | Maternal BDE209 exposure during lactation causes reproductive toxicity in peripubertal mice offspring with down-regulation in GLUT3 and GLUT8 expressions and decreased LDH activity. |

**Table 2.** *Cont*.

| **Authors/**  **Year** | **Animal** | **Exposure** | **Exposure Concentration** | **Main Findings** |
| --- | --- | --- | --- | --- |
| Lin Cheng(44)  2021 | male broilers (Arbor Acres) | BDE209 | 0-4g/kg(42 days) | Oral intake of BDE209 can cause structural injuries and even hyperplasia, and affect gene transcription involved in the neuroactive ligand-receptor interaction pathway of thyroid, and thyroid hormones in serum |
| Debarshi Sarkar(17)  2021 | mice | BDE209 | 500, 700 mg/kg (28 days) | Maternal exposure to BDE209 during lactation causes reproductive toxicity in adult mice offspring. |
| Taotao Liao(38)  2021 | female Balb/c mice | BDE209 | 4, 40, 400mg/kg(20 days) | BDE209 exposure could cause immunotoxicity. That the effect could be dose-dependently reduced after withdrawal of BDE209. |
| Xinpei Wang(30)  2022 | human thyroid cells | BDE209 | 1, 2.5μM (27 days) | BDE209 has chronic toxicity and potential tumourigeniceffects on the thyroid by inhibiting TRß. |
